# *Staphylococcus hominis* subspecies can be identified by SDS-PAGE or MALDI-TOF MS profiles

**DOI:** 10.1038/s41598-019-48248-4

**Published:** 2019-08-13

**Authors:** Eliezer Menezes Pereira, Claudio Simões de Mattos, Olinda Cabral dos Santos, Dennis Carvalho Ferreira, Tamara Lopes Rocha de Oliveira, Marinella Silva Laport, Eliane de Oliveira Ferreira, Katia Regina Netto dos Santos

**Affiliations:** 1 Instituto Federal de Educação, Ciência e Tecnologia do Rio de Janeiro (Campus Pinheiral), Rio de Janeiro, Brazil; 2 Departamento de Microbiologia Médica, Instituto de Microbiologia Paulo de Góes, Universidade Federal do Rio de Janeiro, Rio de Janeiro, Brazil; 3 Faculdade de Odontologia, Universidade Veiga de Almeida, Faculdade de Odontologia, Universidade Estácio de Sá, Rio de Janeiro, Brazil

**Keywords:** Bacteriology, Proteomics, Proteomic analysis

## Abstract

*Staphylococcus hominis* is part of the normal human microbiome. Two subspecies, *S. hominis hominis* (*Shh*) and S*. hominis novobiosepticus* (*Shn*), have clinical significance. Forty-nine *S. hominis* isolates were analyzed by the MicroScan automated system, SDS-PAGE and MALDI-TOF methods, followed by partial sequencing of the 16S rDNA gene. The trehalose fermentation test, disk diffusion and broth microdilution tests were used to identify (novobiocin test) and access the susceptibility to oxacillin and vancomycin of isolates. The SCC*mec* elements and genomic diversity were evaluated by PCR and PFGE methods, respectively. Profiles of 28 (57%; 8 *Shh* and 20 *Shn*) isolates corroborated with the results found in all the applied methods of identification. The remaining 21 (43%) isolates were phenotypically identified as *Shh* by MicroScan; however, they were identified as *Shn* by SDS-PAGE and mass spectral, and confirmed by 16S rDNA sequencing. Among 41 isolates identified as *Shn* by the molecular and mass spectrometry methods, 19 (41%) were novobiocin-sensitive, and the trehalose test indicated 11 positive isolates, which are considered atypical phenotypic results for this subspecies. In addition, 92.7% of the isolates identified as *Shn* by these methods carried *mec*A gene, while only 12.5% of the *Shh* isolates were positive. Together, the results highlighted the SDS-PAGE and MALDI-TOF MS methods as promising tools for discriminating *S. hominis* subspecies.

## Introduction

Coagulase-negative *Staphylococcus* (CoNS) is an important group of gram-positive coccoid bacteria. They are mainly associated with human and animal biota and are also detected in blood cultures of hospitalized patients^1–3^. The main concern with those microorganisms is the possibility of serving as a reservoir for resistance and virulence genes^4^. Among CoNS species, *Staphylococcus hominis* is the third most frequent one, is regarded as a causal agent of bacteremia and endocarditis^2^.

As described amongst staphylococcal isolates, resistance to oxacillin (or methicillin) in *S. hominis* occurs because of the acquisition of the *mec*A gene, which encodes an altered penicillin-binding protein with low affinity to beta-lactam drugs^5^. This gene is located in a mobile genetic element, called Staphylococcal Cassette Chromosome *mec* (SCC*mec*), which is present in *Staphylococcus* genus and is highly conserved among its species^6^. *S. hominis* isolates have also been reported to show decreased susceptibility to vancomycin^7^, as well as resistance to linezolid^8^.

Amongst *S. hominis* isolates, two subspecies have been described: *S. hominis hominis* (*Shh*) and *S. hominis novobiosepticus* (*Shn*). The latter is usually characterized as novobiocin-resistant and unable to form acid aerobically, even by using trehalose or N-acetylglucosamine^9^. In addition, *Shn* clinical isolates are usually described as multiresistant and are frequently isolated in bacteremia cases, compared to other subspecies^2,7,10–12^. However, studies have demonstrated that *Shn* isolates are usually misidentified when only biochemical or automated methods are used^13,14^, making its characterization unreliable. In addition, few studies have shown the genomic diversity of the *S. hominis* subspecies^15,16^, and a reliable standard method to identify the subspecies has not been made available yet. According to Zhang and coworkers^16^, a discrimination of the two subspecies may be difficult since *Shn* seems to form a paraphyletic taxon. However, tools that help in the better characterization of *S. hominis* isolates would contribute to generating more accurate clinical epidemiology data. Based thereon, this work aimed to evaluate different methods to try to improve the discrimination of *Shh* and *Shn* isolated from blood cultures, including their susceptibility to oxacillin and vancomycin and clonal profiles.

## Results

**Figure 1:**
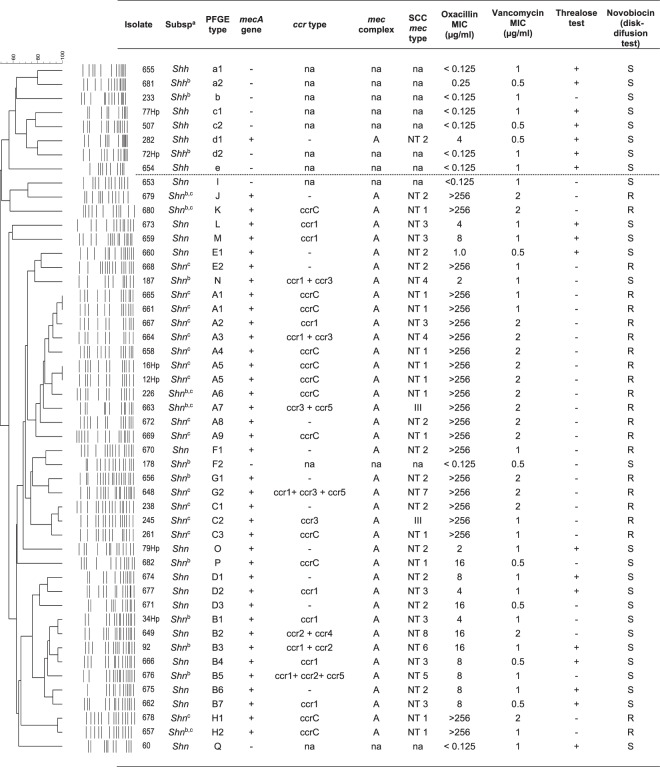
Dendrogram of the pulsed-field gel electrophoresis (PFGE) profiles of *Sma*I and *Apa*I-digested genomic DNA of 49 *Staphylococcus hominis* spp. isolates and associated characteristics. Similarities percentage is identified on dendrogram derived from the unweighted pair group method using arithmetic averages and based on Dice coefficients; +: positive; −: negative; SCC*mec*: Staphylococcal chromosomal cassette *mec*; MIC: minimum inhibitory concentration in µg/mL; oxa: oxacillin; van: vancomycin; novob: novobiocin; na: not-applicable; NT: non-typeable; ^a^subspecies identification based on total proteins profile by SDS-PAGE and MALDI-TOF, and by partial sequencing of the 16S rDNA gene; ^b^isolates submitted to partial rDNA 16S sequencing; ^c^isolates identified as *Shh* by the Microscan automated system; ^d^absence of *ccr* complex.

Forty-nine *S. hominis* isolates were initially identified at subspecies level by phenotypic tests by using the MicroScan WalkAway^®^ automation and novobiocin susceptibility tests. Then, the SDS-PAGE and mass spectrometry (MALDI-TOF MS) methods were also applied for all isolates. All methods were concordant to identify an amount of 28 (57%) isolates, 8 *S. hominis hominis* (*Shh*) and 20 *S. hominis novobiosepticus* (*Shn*) (Groups I and II, Supplementary Material 1). The other 21 (43%) isolates (Groups III and IV) could not be properly discriminated, and by comparing all three methodologies (SDS-PAGE, mass spectrometry methods and by 16S RNA analysis), all were identified as *Shn*. In addition, 19 of these 21 isolates (Group III) were sensitive to the novobiocin disk and presented low minimum inhibitory concentration (MIC) values, ranging from <0.25 to 1.0 µg/ml (Fig. 1). The two remaining isolates (656 s and 670 s from Group IV) were resistant to novobiocin (MICs of 8.0 and 16 µg/mL).

**Figure 2:**
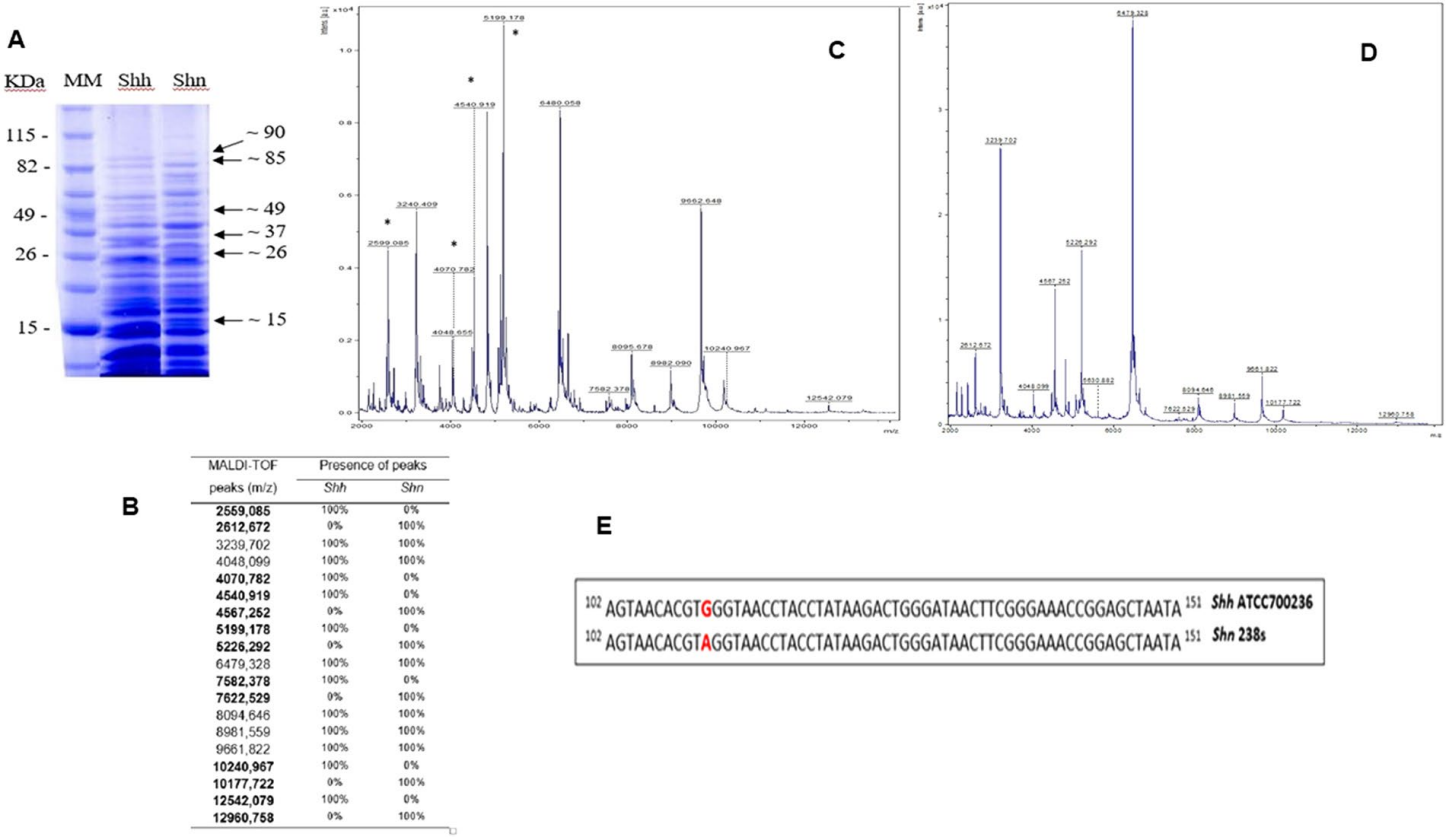
Overview of the mass spectra by MALDI-TOF and proteins profile in SDS-PAGE of *S. hominis hominis* in comparison to the mass spectrum of and *S. hominis novobiosepticus*. (**A**) SDS-PAGE showing the proteins profiles of *S. hominis hominis* (*Shh*) in comparison to the *S. hominis novobiosepticus* (*Shn*). Arrows (→) indicate the prominent proteins for each subspecies; (**B**) Distribution of the detected masses by MALDI-TOF mass according to m/z spectra peaks obtained from *Shh* and *Shn*. Values in bold means spectra peaks found in *Shh* or *Shn*; (**E**) Section of 350 bp amplicon fragment (base 102 to 151) obtained by DNA sequencing showing the Single Nucleotide Polymorphism (SNP 112:G**→**A) of rDNA 16S amplicon, which discriminate *Shh* and *Shn*. **(C,D**) *Each peak represents a different mass (m/z) of an intact protein detected in the analyses for *Shh* or *Shn*.

The SDS-PAGE analysis^14^ showed common regions between *Shh* and *Shn* isolates (similarity index of 90.4%), although proteins with a molecular mass of about 49 kDa and 85 kDa were present only in *Shh*, while *Shn* presented exclusive proteins of about 15 kDa, 26 kDa, 37 kDa and 90 kDa (Fig. 2A). By using the same cell extract, the mass spectrometry analysis also showed a distinct protein profile spectrum, in a range from 2000 m/z to 13000 m/z, between *Shn* and *Shh* strains (Fig. 2B). In fact, based on the data analysis, with the peak list (Presence – 100%; absence – 0%) revealed different proteins peaks between *Shh* and *Shn* (Fig. 2C,D). In both *S. hominis* subspecies the different masses proteins (m/z) were concentrated in the range from 2559 Da to 5226 Da. Figure 2E presents a section of 350 bp amplicon fragment (bases 102 to 151) obtained by DNA sequencing showing the Single Nucleotide Polymorphism (SNP 112: G→A) of rDNA 16S amplicon, which discriminate the subspecies *Shh* and *Shn*. Thus, based on these data, all the 21 misidentified isolates were identified as *Shn*.

Susceptibility to methicillin was evaluated by the detection of the *mec*A gene, and 39 (79.6%) *S. hominis* isolates were positive. Out of the 41 isolates identified as *Shn* by the MALDI-TOF and SDS-PAGE methods (Fig. 1; Supplementary Material 1), 92.7% carried *mec*A gene, while only 12.5% of the *Shh* isolates were positive. The oxacillin MIC ranged from <0.125 to 4 µg/mL for *Shh*, and from <0.125 to >256 µg/mL for *Shn* when the classification by MALDI-TOF and SDS-PAGE was considered. Similarly, vancomycin MIC was also evaluated and the values ranged from 0.5 to 2 µg/mL for *Shn* isolates (identified by MALDI-TOF and SDS-PAGE methods), but 85.4% of them presented MIC from 1 to 2 μg/mL. On the other hand, *Shh* isolates (also identified by MALDI-TOF and SDS-PAGE methods) presented vancomycin MIC values ≤ 1 µg/mL.

Considering the PFGE results, the dendrogram obtained by Bionumerics analysis of the 49 *Shn* isolates, showed no clonal relationship between *S. hominis* subspecies isolates (Less than 50% of similarity). Among *Shh* isolates (PFGE types “a” to “e”) one isolate was positive for the *mecA* gene, but the *SCCmec* complex was non-typeable (NT). The 41 *Shn* isolates were clustered into 17 PFGE types, alphabetically labeled “A” to “Q”, and just two PFGE types (“A” and “B”) comprised 44% of the isolates. Among the *Shn* isolates, 38 (92.7%) were *mecA* positive, and only two showed typeable SCC*mec* elements (type III). The other 36 *mecA* positive *Shn* isolates were included in eight NT SCC*mec* profiles, as it follows: NT1–12 isolates (*ccr*C + *mec* complex A); NT2–11 (*ccr* absent and *mec* complex A); NT3–7 (*ccr*1 + *mec* complex A); NT4–2 (*ccr*1 + *ccr*3 + *mec* complex A); NT5–1 (*ccr*1 + *ccr*2 + *ccr*5 + *mec* complex A); NT6–1 (*ccr*1 + *ccr*2 + *mec* complex A); NT7–1 (*ccr*1 + *ccr*3 + *ccr*5 + *mec* complex A); and NT8–1 (*ccr*2 + *ccr*4 + *mec* complex A).

## Discussion

*Staphylococcus hominis* is the third species of CoNS identified in bloodstream infections^4,17^. This identification is usually based on automated (eg. MicroScan) and/or biochemical tests, which discriminates the species into two subspecies: *S. hominis hominis* (*Shh*) and *S. hominis novobiosepticus* (*Shn*). Resistance to novobiocin and failure to produce acid aerobically from D-trehalose and N-acetyl-D-glucosamine were pointed out as the main distinguishable characteristics of *Shn*, when *S. hominis* subspecies were described in 1998^9^. However, several authors have described how complicated it is to differentiate *S. hominis* isolates into subspecies^18,19^. Recently, a study^16^ aimed to genetically characterize *S. hominis* isolates by using MLST, showing that phylogenetic analyses indicate that *Shn* sequence-types (STs) do not form a single, well-supported cluster, and maybe a paraphyletic taxon. In the present study, we evaluated 49 S. *hominis* isolates from blood obtained at a tertiary hospital in Rio de Janeiro and 28 (57%) of them were concordant with all the identification methods performed. We used the SDS-PAGE method as a tool to discriminate the *S. hominis* subspecies, as recommended previously^14^. We also included a MALDI-TOF MS analysis, in order to obtain a better characterization of these isolates. Our data show that both SDS-PAGE and MALDI-TOF MS analyses helped confirmed MicroScan/Novobiocin resistance tests results for the majority of the isolates, showing they are useful tools to differentiate *S. hominis* subspecies. The MALDI-TOF MS method highlighted 13 specific peaks for *Shn* or *Shh*, and seven of them were discriminatory, which can potentially be used to distinguish these two subspecies. The SDS-PAGE showed six different protein bands, and these subspecies could be visually distinguishable by using reference strains for comparing.

In relation to the other 21 isolates, the results of the phenotypic tests were not concordant with those of the SDS-PAGE and MALDI-TOF MS tests. The MicroScan automated test identified these isolates as *Shh*. However, SDS-PAGE and MALDI-TOF identification showed that these isolates belonged to the *Shn* subspecies. In addition, SDS-PAGE and MALDI-TOF data are in disagreement with the results of the biochemical methods applied (trehalose fermentation and novobiocin resistance tests). Since 19 of these 21 isolates identified as *Shn* by SDS-PAGE and MALDI-TOF were proven to be novobiocin-sensitive, this result suggests/confirms that phenotypical analysis is not reliable to distinguish *S*. *hominis* subspecies, and nor is the trehalose test, which indicated 11 of all *Shn* isolates (identified by the SDS-PAGE and MALDI-TOF MS) as positives for this test. It is recognized that automated methods (e.g. MicroScan) show several inconsistences with regard to bacterial phenotypical characterization, as described previously^20^. The failure of the novobiocin-resistance test has also been described before^18,19^. Zhang and coworkers^16^ described a single-nucleotide polymorphism (SNP) in the *gyrB* gene that encodes a bacterial DNA gyrase, which is the target of novobiocin resistance. They evaluated 108 *S. hominis* isolates, and out of 38 novobiocin-resistant isolates, all of them showed the SNP 431: G→T, resulting in a predicted amino acid polymorphism, 144:R→L. All 70 novobiocin-sensitive isolates had the G431 allele, and all 38 novobiocin-resistant isolates had the T431 allele. These data demonstrated the relation between SNP polymorphism and novobiocin resistance, but not regarding the identification of *S. hominis* subspecies, indicating that this phenotypic test is not useful to discriminate *Shn* and *Shh*, as shown in the present study. The authors used the trehalose degradation test as a discriminatory test to separate both *S. hominis* subspecies. What drew our attention was that 11 (27%) out of 41 *Shn* isolates from our collection were positive for this test. Using the parameters described by Zhang and coworkers^16^ to evaluate our results, 11 isolates of *Shn* would be identified as *Shh* and one isolate of *Shh* would be identified as *Shn*.

In view of the results disclosed herein, chances are that the present study is the first one to use MALDI-TOF MS data to differentiate these subspecies, bringing a higher specificity to *Shh* and *Shn* discrimination. MALDI-TOF MS is believed to be the future of bacterial identification, in view of its high specificity and short analytical time. In addition, it is an excellent tool for identifying pathogens and it inaugurates a new era in modern microbiology^21^. In order to confirm our protein profile data, 15 isolates from different phenotypical groups had their rDNA 16S sequenced, and the results were found to be concordant with the MALDI TOF MS analyses.

In Brazil, some studies have reported oxacillin resistance rates of 86% among CoNS isolated from bacteremia^22,23^. Evaluating the resistance rates between *S. hominis* subspecies, Palazzo and colleagues^10^ found 83.3% of oxacillin resistance among *Shn* isolates, and Caierão and coworkers^24^ detected oxacillin resistance among all *Shn* isolates. In the present work, 97.4% of the *mec*A positive isolates were identified as *Shn* and the majority of chromosome cassettes were non-typeable, as already described^25^. The detection of oxacillin/methicillin resistance is extremely relevant since its early and accurate observation can assist in establishing the correct antibiotic therapy to combat infection. In our study, a high prevalence of oxacillin resistance was observed among the *Shn* isolates (92.7%), while only 12.5% of the *Shh* isolates were oxacillin-resistant, which confirms the existence of higher resistance rates among the isolates of the *Shn* subspecies. The *Shn* isolates also expressed higher MICs to vancomycin (85.4% presented MIC from 1 to 2 µg/mL), as already described by Sorlozano and coworkers for this subspecies^26^. On the other hand, all *Shh* isolates had vancomycin MIC ≤ 1 µg/mL. Therefore, we may conclude that an analysis of the total protein profile, by SDS-PAGE or MALDI-TOF MS, of *S. hominis* subspecies together with the evaluation of their oxacillin and vancomycin susceptibilities, could provide us with better discrimination between the *Shh* and *Shn* subspecies.

Studies in the literature concerning CoNS genomic diversity describe the high genomic diversity of isolates, including the *S. hominis* subspecies^2^. We analyzed the *S. hominis* isolates by PFGE to determine the different PFGE types, and each subspecies was grouped independently and the dendrogram showed no clonal relationship between them. Among *Shh* isolates, related profiles were found. For 41 *Shn* isolates, despite the clonal diversity, 44% of the isolates belonged to only two clonal groups (“A” and “B”). Moreover, it should be noted that the isolates of genotype “A” (subtypes A1, A1, A2, A4 and A8) were obtained in the same period from the same hospital (data not shown), confirming the ability of this *S. hominis* subspecies to spread and stay in this setting.

In conclusion, our results showed that the phenotypical methods normally used for discrimination of *S. hominis* subspecies are not reliable. Hence, we suggest that protein profile analysis and/or MALDI-TOF MS analysis might be complementary and useful tools to discriminate these subspecies more accurately, contributing to understanding its clinical epidemiology.

## Methods

### Bacterial isolates and reference strains

Forty-nine *S. hominis* isolates, obtained from blood cultures at a tertiary care hospital located in Rio de Janeiro, Brazil, and belonging to our culture laboratory collection, were used for this study. The hospital is an institution with 532 beds, 35 of which are in an Intensive Care Unit (ICU). All isolates were collected between 1998 and 2002 (17 isolates); and from 2005 to 2009 (32 isolates). One ATCC 27844 *S. hominis hominis* and a clinical isolate 34Hp *S. hominis novobiosepticus*^14^ were included as control strains. ATCC 29213 *S. aureus* and ATCC 33591 strains were also included as controls for the methicillin and vancomycin susceptibility tests.

### Isolates identification

All *S. hominis* isolates were initially identified phenotypically by the Microscan WalkAway automated system (Dade Behring Microscan Inc. Division, West Sacramento, USA). Additional tests were performed as follows:

### Novobiocin susceptibility and trehalose tests

Novobiocin (Sigma-Aldrich Company, St. Louis, USA) susceptibility determination by using the disk diffusion and broth microdilution methods was performed in accordance with Bannerman and Peacock (2007)^27^ and the Clinical and Laboratory Standards Institute (CLSI)^28^. Concentration ranged from 0.250 to 16 µg/mL. Acid production from D-trehalose was determined according to Iorio and coworkers^13^

### Protein profile analysis

In order to discriminate *Shh* from *Shn* isolates, whole-cells protein extracts were prepared according to Santos and coworkers^14^. Briefly, to obtain the protein extracts the cells were cultured overnight until they reached a concentration of 10^7^ CFU/mL Cells were collected by centrifugation at 12,000 × *g* for 5 min. After being washed twice in 0.85% NaCl, the cells were incubated in the presence of 0.2 mg/mL lysostaphin for 2 h at 37 °C. After the incubation period, equal volumes of 0.5 M Tris-HCl (pH 7.2) buffer containing 4% SDS, 10% Beta-mercaptoethanol, 20% glycerol, and 0.1% bromophenol blue were added, and the samples were boiled for 5 min. Cellular extracts were submitted to Sodium Dodecyl Sulfate Polyacrylamide Gel Electrophoresis (SDS-PAGE) analysis. The obtained band patterns were analyzed through the Bionumerics program, version 6.0 (Applied Maths, Belgium), and similar percentages were derived from the unweighted pair group method using arithmetic averages and based on Dice coefficients.

### Maldi-tof Ms

Mass spectrometry technology through the Matrix Assisted Laser Desorption Technology/Ionization Time of Flight Mass Spectrometry (MALDI-TOF MS) was used in accordance with Alatoom and coworkers^29^ to identify *S. hominis* subspecies. All strains of the groups III and IV, seven of the group II and two of the group I were used to construct the database (Supplementary Material 1). Two reference strains were used: 238 s (*Shn*) and 72 Hp (*Shh*) (Santos *et al*., 2009). Five bacterial colonies of isolates previously identified as *Shh* and *Shn* by SDS PAGE method were dissolved in 300 µL of HPLC water (Tedia) in microtubes and mixed. Then, 900 µL of ethanol P.A. (Sigma-Aldrich) was added and it was vigorously homogenized. The suspension was then centrifuged at 16,000 × g for 2 min and the supernatant discarded. The pellet was allowed to dry at room temperature for approximately 20 min. After dried, 20 µL of 70% formic acid (Sigma-Aldrich) and 20 µL of acetonitrile P.A. (Sigma-Aldrich) was added to the pellet and gently homogenized, centrifuged and the supernatant transferred to a differently labelled microtube for further MS analysis. All tubes were kept at −80 °C for two days until the moment of use and 10 days maximum

For the mass spectrometry analysis, 1 µL of the extraction solution was added onto the spot a MALDI-TOF MS plate (MSP 96 target polished steel BC, Bruker Daltonics). Samples were allowed to dry and each spot was covered with 1 µL of 10 g/L CHCA matrix (Cyano-4-hydroxycinnamic acid (Bruker Daltonics) in 50% acetonitrile (Tedia) and 2.5% trifluoroacetic acid (Tedia). For every isolate, 27 spots were applied to produce 27 technical replicates of spectra. Spectra were obtained on Microflex LT mass spectrometer (Bruker Daltonics) and the FlexAnalysis software was used for default parameters (laser frequency of 60 Hz, ion source voltages of 2.0 and 1.8 kV, and lens voltage of 6 kV), generating spectra in a range of 2,000–20,000 m/z. All spectra from Biotyper were preprocessed and normalized using standard parameters (baseline subtraction by running ruling disc and peak detection using a signal to noise ratio of 10). After the preprocessing, a peak match was performed using a tolerance of ±0.002 m/z. Subsequently, by using the MALDI Biotyper 3 software (flex control and flex analysis software) the 27 spectra were compiled to form a single spectrum and the most prevalent and with the highest masses (m/z) were considered the ideal fingerprint spectrum to differentiate the subspecies.

### rDNA single nucleotide polymorphism analysis

To discriminate *Shh* from *Shn*, 15* S. hominis* isolates from different phenotypical groups were selected and, a partial sequencing of the 16S rDNA gene, which includes a single nucleotide polymorphism (SNP), was performed^14^. For the analysis, primers were designed by using the Oligo Analyzer (Integrated DNA Technologies, Coralville, IA, USA) and BioEdit (Ibis Biosciences, Carlsbad, CA, USA), based on the GenBank sequences database (Accession Numbers: KU364060; MG255965; MF327693 for *Shh*, and MG201782; KY218859 for *Shn*). The following primers were designed to amplify a 350 bp fragment of *S. hominis* 16S rDNA gene.: SH16S-F (5′ GCTTGCTCCTTTGACGTTAG 3′) and SH16S-R (5′ CGAAGACCTTCATCACTCAC 3′). Oligonucleotides were purchased from Integrated DNA Technologies, Inc. (Coralville, IA, USA).

DNA was obtained according Walsh, Metzer & Higuchi^30^ as previously described. Amplification was performed on a Programmable Thermal Controller (Eppendorf Mastercycler Gradient, Hamburg, Germany) with the following conditions were: denaturation for 5 min at 94 °C, followed by 35 cycles of 94 °C for 1 min, 58 °C for 1 min and 72 °C for 1 min, with a final extension at 72 °C for 7 min. Amplified products were analyzed on 1% agarose gels, stained with UniSafe (Uniscience, São Paulo, Brazil) and visualized on a UV transilluminator. PCR band products of each gene were purified by using GTX PCR, according to the manufacturer’s instructions (GE 50 Healthcare, Buckinghamshire, England). Sequencing was made in an automated DNA Mega BACE1000 (Biotech, EUA), using the DYEnamic ET Dye Terminator system (GE Healthcare, England). Both DNA strands (forward and reverse) were sequenced and the reverse one was analyzed. To identify the previously mentioned mutation point (112: G→A), sequences obtained were analyzed by using Bioedit (Carlsbad, USA).

### Oxacillin and vancomycin MIC tests

Minimum inhibitory concentrations (MICs) to oxacillin and vancomycin (Sigma-Aldrich Company) were determined by broth microdilution tests according to CLSI 2012^28^. The concentrations ranged from 0.125 to 256 µg/mL for oxacillin and from 0.125 to 16 µg/mL for vancomycin. The ATCC strain 29213 was used as control for the MIC tests.

### Detection of *mec*A gene, SCC*mec* and PFGE typing

The methodology used for the *mecA* gene detection and SCC*mec* typing were performed according to Del Vecchio and coworkers^31^ and Kondo and coworkers^32^, respectively. After DNA extraction^33^ the SCC*mec* typing was performed through two multiplex PCR reactions to detect the *ccr* complex and the *mec* gene class. The combination of the types of *ccr* and *mec* class allowed the identification of the types of SCC*mec* (I to VI).

All isolates were typed by pulsed-field gel electrophoresis (PFGE) after digesting whole cell DNA with *Sma*I and *Apa*I to obtain specific fragments^26^ in a CHEF-DRIII system (Bio-Rad, Richmond, CA, USA), as previously described^34^ with some modifications. Briefly, the *S. hominis* immobilized DNA in agarose blocks were transferred to a solution containing 10 U of *Apa*I (Biolabs), supplemented with 1.5 µL of BSA (Bovine Serum Albumin) at 10 mg/mL, for 18–24 h at 25 °C, followed by incubation with *Sma*I (4 h at 25 °C). To determine the similarity index between the isolates the PFGE fingerprints were analyzed by the BioNumerics program version 6.0 (Applied Maths, Belgium) using the Dice correlation coefficient and the unweighted pair-group method with arithmetic mean (UPGMA) clustering analysis. Isolates with four or fewer bands of difference or a minimum of 80% similarity were designated as the same genotype^35^.

## Supplementary information

Supplementary material 1
