# Supplementary material for: Staphylococcus hominis subspecies can be identified by SDS-PAGE or MALDI-TOF MS profiles
Source: Sci Rep. 2019 Aug 13;9:11736. doi: 10.1038/s41598-019-48248-4 (PMC6692339; doi:10.1038/s41598-019-48248-4)
Supplement: Supplementary file 1 — Supplementary material 1 [file 41598_2019_48248_MOESM1_ESM.pdf]

Title: ***Staphylococcus hominis* subspecies can be identified by SDS-PAGE or MALDI-TOF MS profiles**

Eliezer Menezes Pereira[1,2]; Claudio Simões de Mattos[2]; Olinda Cabral dos Santos[2]; Dennis Carvalho Ferreira[3]; Tamara Lopes Rocha de Oliveira[2]; Marinella Silva Laport[2]; Eliane de Oliveira Ferreira[2] and Katia Regina Netto dos Santos[2,*]

Instituto Federal de Educação, Ciência e Tecnologia do Rio de Janeiro (Campus Pinheiral)[1]; Departamento de Microbiologia Médica, Instituto de Microbiologia Paulo de Góes, Universidade Federal do Rio de Janeiro[2]; Faculdade de Odontologia, Universidade Veiga de Almeida, Faculdade de Odontologia, Universidade Estácio de Sá, Rio de Janeiro, Brazil[3]

Supplementary material 1 – Identification tests and presence of *mec*A gene in subspecies among 49 *Staphylococcus hominis* isolates

Group of isolates	Isolates number (n)	Identification tests					Presence of *mecA* gene (n)
		MicroScan automated system	Novobiocin test		SDS-PAGE total proteins	Mass spectra by MALDI-TOF MS	
			0.04U disk	MIC or range (µg/ml)			
**I**	**72Hp,** 77Hp, 233s, 507s 654s, **655s**, 282s, 681s (8)	*Shh*	S	< 0.25	*Shh*	*Shh*	negative (7) positive (1)
**II**	**12Hp**, 16Hp, 226s**, 238s**, 245s, 261s, 648s, **657s,** 658s, **661s**, **663s**, **664**s, 665s, 667s, 668s, 669s, 672s, 678s, 679s, **680s** (20)	*Shn*	R	> 16	*Shn*	*Shn*	Positive
**III**	**178s, 60s, 79Hp, 92s, 659s, 666s, 673s, 674s, 675s, 676s, 677s, 34Hp, 187s, 649s, 653s, 660s, 662s, 671s, 682s** (19)	*Shh*	S	< 0.25 - 1.0	*Shn*	*Shn*	positive (16) negative (3)
**IV**	**656s, 670s** (2)	*Shh*	R	8.0 - > 16	*Shn*	*Shn*	positive

Shh – *S. hominis hominis*; *Shn* – *S. hominis novobiosepticus*; MIC – minimum inhibitory concentration; the isolates in bold were used to construct the library in the MALDI-TOF MS.
